# Patient reported outcome measures for Spanish-speaking adults with bronchiectasis: systematic review of measurement properties

**DOI:** 10.1007/s11136-025-04130-7

**Published:** 2025-12-23

**Authors:** Yasmina Hamam-Alcober, Cristina Cimarras-Otal, Juan Antonio Sáez-Pérez, Beatriz Herrero-Cortina

**Affiliations:** 1 Universidad San Jorge, Zaragoza, Spain; 2 Precision Medicine in Respiratory Diseases Group, Health Research Institute of Aragon (IIS Aragón), Zaragoza, Spain

**Keywords:** Patient-reported outcome measures, Quality of life, Symptoms, Bronchiectasis, Spanish

## Abstract

**Purpose:**

Patient reported outcome measures (PROMs) are key tools for monitoring and evaluating treatment effectiveness in people with bronchiectasis. However, most are developed for Anglophone contexts, limiting their applicability to non-English-speaking populations. This systematic review aimed to evaluate the measurement properties of PROMs for Spanish-speaking individuals with bronchiectasis.

**Methods:**

A search of major databases was conducted up to August 2024, targeting studies that assessed the measurement properties (validity, reliability, and responsiveness) of any PROMs available for Spanish-speaking adults with bronchiectasis. The methodological quality of the included studies, as well as the quality of the measurement properties was evaluated according to the COSMIN (Consensus-Based Standards for the Selection of Health Status Measurement Instruments) guidelines.

**Results:**

Of 3752 articles, four studies were included. The PROMs were the Leicester Cough Questionnaire (LCQ), Quality of Life Questionnaire for Bronchiectasis (QoL-B), COPD Assessment Test (CAT), and St. George’s Respiratory Questionnaire (SGRQ).Content validity was rated as sufficient with low or very low-quality evidence. Structural validity was assessed only for the SGRQ, rated inadequate with very low evidence. Cross-cultural validity could not be evaluated. Convergent validity was sufficient for all PROMs, highest for LCQ and QoL-B. Internal consistency was indeterminate across PROMs, though limited by lack of structural validity. Test–retest reliability was high for LCQ and moderate for QoL-B and CAT. Responsiveness was sufficient for all three PROMs assessed, with evidence quality from very low to moderate.

**Conclusion:**

Few PROMs exist for Spanish-speaking adults with bronchiectasis. Content, structural and cultural validity, and responsiveness are the least studied properties, limiting treatment monitoring and assessment.

**Registration:**

PROSPERO International register of systematic reviews, CRD42023388173.

## Background

Bronchiectasis is a chronic respiratory disease characterised by a complex pathogenetic intersection involving not only airway inflammation but also mucociliary dysfunction and infection, resulting in permanent bronchial dilatation [1, 2]. The prevalence of bronchiectasis demonstrates gender disparities and increases with age, while also exhibiting geographical heterogeneity [3]. This condition presents a significant challenge to healthcare systems due to its high disease-related resource utilization and associated costs, which can exceed those of matched healthy individuals [4, 5] and it is a pattern observed worldwide, including Spanish-speaking countries [6].

Patients with higher disease activity are characterised by an excessive inflammatory response, often experience intense and persistent symptoms, as well as a high frequency of exacerbations [1, 2]. Among these symptoms, daily cough, chronic sputum production, rhinosinusitis and dyspnoea are particularly frequent [7]. In addition to respiratory symptoms, people with bronchiectasis frequently exhibit extrapulmonary manifestations, including reduced exercise capacity, pain, fatigue, and lifestyle changes such as increased sedentary behaviour [8, 9]. These diverse symptoms have a multidimensional impact on patients, affecting their physical, psychological and social well-being, and ultimately worsening health-related quality of life [8, 10].

Assessing symptom burden in bronchiectasis is a crucial factor in identifying patients with high disease activity and an elevated risk of accelerated disease progression [1, 2]. The use of “Patient Reported Outcomes Measures” (PROMs) is widely recommended to evaluate the mode and magnitude in which these manifestations interference patient´s daily life [11]. PROMs have emerged as essential tools in both research and clinical management of bronchiectasis [12, 13]. These instruments provide healthcare professionals with valuable insights into patients' perspectives regarding their health outcomes, specifically in relation to symptoms, functionality, health behaviours and satisfaction with treatment and healthcare services [14]. A key advantage of PROMs is their ability to detect changes that are meaningful to the patient following treatment or disease progression [15, 16]. This feature enables the application of personalized therapies and makes PROMs essential for longitudinal patient monitoring in clinical settings.

The incorporation of PROMs as relevant outcome measures in bronchiectasis research is increasing, as they provide a patient-centered perspective that does not always align with objective clinical measures [17]. Indeed, studies in bronchiectasis have shown that patients with higher symptom burdens, as measured by PROMs, are more responsive to interventions [15, 16]. Therefore, to achieve an integrative and global assessment, patients' perspectives must be considered alongside other clinical outcomes.

To ensure their usefulness and the accuracy of the findings, PROMs must undergo rigorous examination of their measurement properties [18]. Although PROMs specifically validated or developed for bronchiectasis were scarce in the past, the field has made significant progress. The predominant PROMs used in bronchiectasis exhibit acceptable measurement properties in their original English versions [12, 13]. According to Consensus-based Standards for selection of health Measurement Instruments (COSMIN) guidelines [18, 19] before implementing a PROM in a population different from the initial validation cohort, a rigorous translation and validation process is necessary. This requirement limits the PROMs application among non-English speaking patients, resulting in disparities in the availability of assessment resources. Therefore, to enhance our understanding of the measurement properties of PROMs applicable to Spanish-speaking patients with bronchiectasis and encourage their use in clinical practice and future research in Spanish-speaking countries, a comprehensive systematic review was conducted.

## Methods

This systematic review was conducted in accordance with the updated COSMIN methodology for systematic reviews of PROMs [18] and was reported following the PRISMA-COSMIN guidelines for systematic reviews of outcome measurement instruments [19]. The protocol was prospectively registered on PROSPERO International register of systematic reviews (CRD42023388173).

### Searches methods and study selection

An electronic database search was conducted in PubMed, the Cochrane Library, Embase and EBSCO from inception to August 2025. The search strategy was conducted using the keyword 'bronchiectasis', in combination with additional terms related to 'patient-reported outcomes', 'quality of life', 'questionnaires', and 'measurement properties', among others. Boolean operators 'AND' and 'OR' were applied to refine and optimize the search. A detailed description of the search strategy is available outlined in Table S1 (supplementary material).Reports obtained from the search strategy were uploaded to Rayyan software for the study selection process. After removing duplicate entries, two independent reviewers (YH-A and CC-O) screened the titles and abstracts, followed by a full-text screening of the remaining articles. To further enhance the study selection process, the reference lists of the included studies were also examined to identify additional relevant studies. Disagreements between the reviewers were resolved by discussion. If consensus could not be reached, a third reviewer (BH-C) was consulted.

### Eligibility criteria

Studies were included if they met two criteria: (1) they recruited adults (≥ 18 years) with bronchiectasis confirmed by high-resolution chest computed tomography and (2) they assessed measurement properties of PROMs that had undergone a translation process specifically involving Spanish-speaking populations [18]. Studies were required to report at least one measurement property: validity, reliability, or responsiveness. The COSMIN taxonomy was applied to evaluate these properties, ensuring consistency in terminology across the studies. All study designs were accepted except for qualitative studies, and abstracts. Studies involving mixed chronic respiratory diseases were excluded unless they provided separate data for bronchiectasis participants.

### Data collection procedure

Data extraction was performed by two researchers (YH-A, CC-O) using a standardised table based on COSMIN guidelines. This table included four main sections: (1) basic study information (e.g., publication year, first author, country); (2) characteristics of the study sample (e.g., sample size, gender distribution, mean age, disease severity, clinical status, and selection criteria); (3) characteristics of the original PROMs evaluated (e.g., original target population and measurement properties); and (4) results of the measurement properties of the Spanish-speaking population validation of the PROMs included.

Content validity is one of the measurement properties evaluated for the PROMs, involving the assessment of relevance, comprehensiveness, and comprehensibility using the 10 COSMIN criteria [20]. In addition to synthesizing available evidence from studies involving patients and health care professionals, the reviewers independently rated the content validity of each PROM against these criteria. Beyond content validity, structural, cross-cultural, convergent, and discriminant domains for validity were also evaluated. Reliability was examined through internal consistency, test–retest reliability, and measurement error. Responsiveness was assessed by comparisons with other measurements, group comparisons, and pre-post intervention assessments. Additionally, the estimation of minimal important difference (MID) values was examined to determine thresholds for clinically meaningful change.

### Risk of bias, rating and quality of the measure assessment

Two independent reviewers (YH-A and BH-C) assessed the methodological quality of the included studies using the COSMIN Risk of Bias checklist (RoB), specifically designed for systematic reviews of PROMs [21].

The translation and cross-cultural adaptation were firstly assessed qualitatively, focusing on three main aspects: the translation process, involvement of professionals, and participation of patients. Specifically, for the validity evaluation, we used targeted questions related to content validity, structural validity, cross-cultural validity, and hypothesis testing (convergent and discriminant validation) from box 2, 3, 5 and box 9. To evaluate the reliability measurement property, we used specific questions from boxes 4, 6 and 7 focusing on internal consistency, test–retest reliability, and measurement error. To assess responsiveness, we utilised questions from box 10 that focused on three key aspects: the relationship between the PROMs's responses and those of similar instruments, the ability to detect differences in responses between groups, and the sensitivity to change following an intervention or disease progression [21]. When formal statistical testing for a given measurement property or domain (e.g., multiple-group confirmatory factor analysis or differential item functioning for cross-cultural validity) was not reported in the included studies, the assessment was restricted to a qualitative description of the methods used.

Each item on the box described was rated using one of four categories: "very good," "adequate," "doubtful," "inadequate" or “not applicable”. Following the updated COSMIN “worst score counts” principle, the overall risk of bias rating for a measurement property was determined by the lowest rating among its items [18]. The final classification used the following labels: sufficient (+) when the criteria for a good measurement property, as defined in the updated COSMIN guidelines, were met; insufficient (−) when these criteria were not met; and indeterminate (?) when data were insufficient or not reported to allow a judgment (Table S2) [20].

For construct validity and responsiveness, the research team formulated a priori hypotheses regarding the expected direction and magnitude of change (Table S3). If at least 75% of the results were consistent with these predefined hypotheses, the overall rating was classified as sufficient; otherwise, it was rated as insufficient. In cases of inconsistent findings, the final judgment was based on the majority of results or supported by clinical reasoning [20].

The independent reviewers (YH-A and BH-C) graded the quality of evidence for each measurement property per each PROMs using the COSMIN-modified Grading of Recommendations Assessment, Development, and Evaluation (GRADE) approach [20]. They considered four key factors: RoB, inconsistency, imprecision, and indirectness. Based on this assessment, the quality of evidence was rated as “high,” “moderate,” “low,” or “very low” [18]. Reasons for downgrading the quality of evidence are reported in the Table S4.

## Results

### Study selection and characteristics

**Fig. 1:**
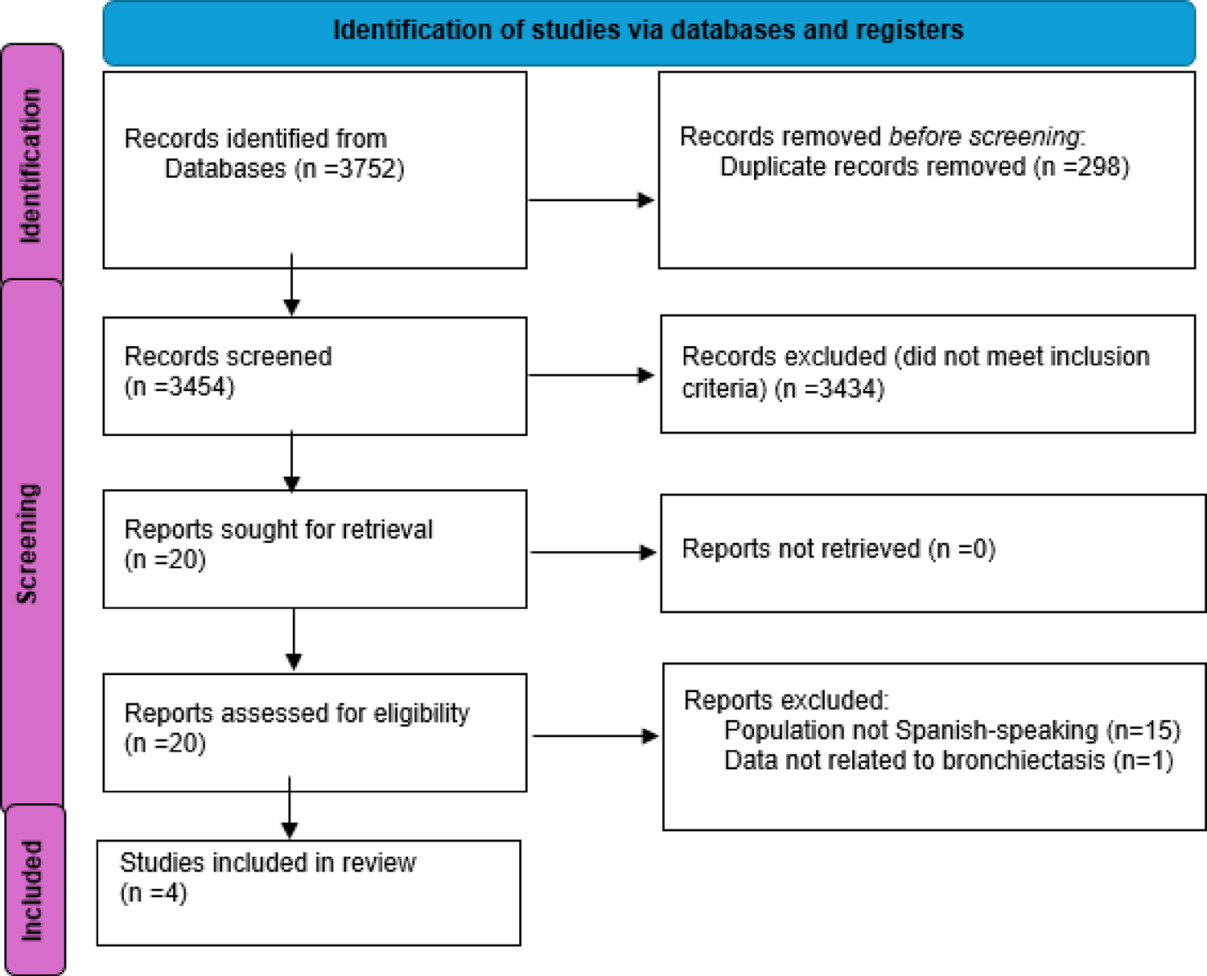
PRISMA flowchart

A total of 3752 records were identified for this systematic review. After removing duplicates, (n = 298), 3454 records remained. Following abstract and title screenings, 3434 records were excluded, leaving 20 for full-text review. No additional studies were found through reference list checks of these full-text articles. Of the 20 full-text reports, 16 were excluded for not meeting the selection criteria as they did not involve Spanish-speaking population (n = 15) or reported data from a mixed population with various respiratory diseases (n = 1). Ultimately, four studies (32–35) were deemed eligible for inclusion in this review. Figure 1 illustrates this screening process based on the PRISMA flowchart format.

The four included studies [22–25] were conducted in Spain between 2005 and 2020. In total, 664 participants were recruited for the studies, with sample sizes ranged from 96 to 259 participants. Females constitute 60% of the overall sample. The mean age of participants across studies varied from 57 to 70 years. All participants had bronchiectasis diagnosed by high-resolution computed tomography and were clinically stable at the time of recruitment.

Two studies [23, 24] primarily evaluated disease severity using validated scoring scales in bronchiectasis, the BSI, the FACED score, and the e-FACED score. Globally, participants were classified into severity categories as follows: 33% to 69% were mild, 25% to 39% were moderate, and 2% to 24% were severe. The remaining two studies [22, 25] included individual outcomes linked to a worse prognosis in bronchiectasis (e.g., lung function, exacerbations) as factors associated with increased severity in the participants. Table S5 (supplementary material) summarises the main characteristics of the included studies and their participants.

### Description of PROMs

The four identified PROMs [22–25] are health-related quality of life questionnaires (the Leicester Cough Questionnaire -LCQ, Quality of Life Questionnaire for Bronchiectasis—QoL-B, COPD Assessment Test -CAT and St. George’s Respiratory Questionnaire -SGRQ) and all were initially developed for English-speaking people. Of the four PROMs, only one was specifically designed for people with bronchiectasis [22]. The remaining three PROMs were originally developed for COPD [24, 25] or chronic cough [23], and were subsequently adapted for use in people with bronchiectasis. While QoL-B, CAT, and SGRQ evaluate multiple symptoms, the LCQ specifically emphasizes the impact of cough on a patient's quality of life. The scoring interpretation varies among these PROMs: higher scores reflect better quality of life in two PROMs [22, 23], while lower scores indicate better quality of life in the others [24, 25]. Three PROMs provide a global score [23–25], and three offer individual domain scores (e.g., physical) [22, 23, 25] (Table 1).

**Table 1:** Description of the patient-reported outcome measures for Spanish-speaking people with bronchiectasis

PROM	Original objective	Items	Domains	Type of questions	Administration	Total scores	Interpretation
LCQ	To evaluate the impact of chronic cough	19	Three: Physical Psychological Social	Likert-type responses ranging from 1 to 7	Self-administration	Each domain and total scores: from 3 to 21	Higher scores = lower impact of cough in daily life
QoL-B	To evaluate quality of life in bronchiectasis	37	Eight: Physical Functioning Role Functioning Vitality Emotional Functioning Social Functioning Treatment burden Health Perceptions Respiratory symptoms	Likert-type and multiple choice	Self-administration	Scores for each domain: from 0 to 100	Higher scores = better health-related quality of life
CAT	To stablish the symptoms severity in COPD	8	No	Likert-type responses ranging from 0–5	Self-administration	Total score from 0 to 40	Higher scores = greater symptom severity
SGRQ	To evaluate quality of life for asthma and COPD	50	Three: Symptoms Activity Impacts	Multiple choice questions and Yes/No questions	Self-administration	Each domain and total scores: from 0 to 100	Higher scores = more impairment quality of life

LCQ, Leicester Cough Questionnaire; QoL-B, Quality of Life Questionnaire for Bronchiectasis; CAT, COPD Assessment Test; SGRQ, Saint George's Respiratory Questionnaire

### Measurement properties of the PROMs

#### Cross-cultural adaptation procedure

While the LCQ and QoL-B studies provided details about the translation process [23, 24], the CAT and SGRQ studies used an existing version that had been previously translated for Spanish-speaking individuals with COPD. Since the authors did not report specific information about the translation adaptation process in these studies, this information was extracted from the original studies evaluating the Spanish translations of these PROMs in COPD [26, 27].

Overall, all PROMs underwent a translation and adaptation process that included both forward and backward translations conducted by bilingual speakers, followed by a review conducted by a professional committee. Additionally, cognitive interviews were conducted with patients to facilitate cultural adaptations.

#### Validity

Data obtained from patients and healthcare professionals regarding content validity were summarised together in Table 2, as the information was identical for both groups across all PROMs. The LCQ and SGRQ incorporated data from interviews with both healthcare professionals and patients, as well as pilot testing with patients. In contrast, the QoL-B appeared to have undergone only pilot testing with patients. However, the available information was insufficient to fully assess relevance, comprehensiveness, and comprehensibility for any of these PROMs from either the healthcare professional or patient perspective. No content validity data were reported for the CAT.

**Table 2:** Results on content validity of patient-reported outcome measures for Spanish-speaking people with bronchiectasis

PROMs	Content validity (patient / HCP)									Content validity (authors´ opinion)				Content validity (overall)							
	n		Relevance		Comprehensiveness		Comprehensibility		Globalrating	Relevance	Comprehensiveness	Comprehensibility	Global rating	Relevance		Comprehensiveness		Comprehensibility		Global	
			RoB	Rating	RoB	Rating	RoB	Rating						Rating	QoE	Rating	QoE	Rating	QoE	Rating	QoE
LCQ	Interviews = 6 Pilot tested = 20	Patient	D	(?)	D	(?)	D	(?)	(?)	(+)	(+)	(+)	(+)	(+)	Low^&^	(+)	Low^&^	(+)	Low^&^	(+)	Low^&^
		HCP	D	(?)	D	(?)	D	(?)	(?)												
QoL-B	Pilot tested = 20	Patient HCP	D	(?)	D	(?)	D	(?)	(?)	(+)	(+)	(+)	(+)	(+)	Low^&^	(+)	Low^&^	(+)	Low^&^	(+)	Low^&^
			D	(?)	D	(?)	D	(?)	(?)												
CAT	NR	Patient HCP					(+)	(+)	(+)	(+)	(+)	Very low^@^		Very low^@^		Very low^@^	(+)	Very low^@^			
SGRQ	*Interviews = 8 *Pilot tested = 23	Patient	D	(?)	D	(?)	D	(?)	(?)	(+)	(+)	(±)	(+)	(+)	Low^&^	(+)	Low^&^	(+)	Low^&^	(+)	Low^&^
		HCP	D	(?)	D	(?)	D	(?)	(?)												

PROMs: patient-reported outcome measures; LCQ: Leicester Cough Questionnaire; QoL-B: Quality of Life Questionnaire for Bronchiectasis; CAT: COPD Assessment Test; SGRQ: Saint George’s Respiratory Questionnaire; HCP, health care professionals; RoB, risk of bias; QoE, quality of evidence; NR, not reported; *: Data were obtained from the original translation study (Ferer M et al. Eur Respir J. 1996 Jun;9(6):1160–6). RoB: Risk of Bias; V: very good; A: Adequate; D: Doubtful; I: Inadequate; (+) sufficient; (−) insufficient; (?) indeterminate; & One content validity with doubtful quality; @: Ratings are only based on authors´opinion

Complementarily, the authors’ subjective evaluations, based on the 10 COSMIN criteria for content validity, were incorporated to supplement the available evidence (Table S6, supplementary material). Overall, all PROMs were rated as sufficient for content validity, primarily according to the authors’ consensus ratings. The quality of evidence was subsequently downgraded to low for the LCQ, SGRQ, and QoL-B, and to very low for the CAT (Table 2).

Structural validity was assessed only for the SGRQ using an exploratory factor analysis (EFA) approach. The analysis extracted four factors, which together accounted for 36.8% of the total variance. The risk of bias was rated as inadequate, and the overall structural validity was judged insufficient, with the quality of evidence graded as very low. Formal analysis of cross-cultural validity was not possible because the included studies did not report relevant statistical testing methods, such as multiple-group confirmatory factor analysis or differential item functioning (Table 3).

**Table 3:** Results on structural and cross-cultural validity of patient-reported outcome measures for Spanish-speaking people with bronchiectasis

PROMs	Structural validity					Cross-cultural validity				
	n	Synthesised results	RoB	Rating	QoE	n	Synthesised results	RoB	Rating	QoE
LCQ	NR					NR				
QoL-B	NR					NR				
CAT	NR					NR				
SGRQ*	102	4 factors explain 36.8% of total variance (EFA method) Factor 1: Activity + physical impact items (19.9%) Factor 2: Symptoms + impact items (6.6%) Factor 3: Impact + activity (5.8%)—low discrimination Factor 4: Impact + activity (4.5%) low discrimination	I	(−)	Very low^@^	NR			–	

PROMs, patient-reported outcome measures; LCQ, Leicester Cough Questionnaire; QoL-B, Quality of Life Questionnaire for Bronchiectasis; CAT, COPD Assessment Test; SGRQ, Saint George’s Respiratory Questionnaire; NR, not reported; EFA, exploratory factor analysis; *: Data were obtained from the original translation study (Ferer M et al. Eur Respir J. 1996 Jun;9(6):1160–6). RoB: Risk of Bias; V: very good; A: Adequate; D: Doubtful; I: Inadequate; (+) sufficient; (−) insufficient; (?) indeterminate. @: -3 Risk of Bias extremely serious

Hypothesis testing was evaluated in all studies [22–25], including convergent and discriminative validity. The SGRQ was the predominant comparator instrument for assessing convergent validity. The CAT study expanded on this approach by incorporating additional measures, specifically the QoL-B instrument and the Bronchiectasis Health Questionnaire (BHQ) [28]. All studies using SGRQ instrument as gold comparator demonstrated sufficient convergent validity and classified as very good according to the RoB checklist. In contrast, the SGRQ study [25] assessed convergent validity by evaluating correlations between SGRQ scores and clinical variables, rather than using another PROM instrument. Consequently, this study was rated as insufficient for convergent validity and classified as inadequate following the RoB checklist. Overall, the methodological quality of the convergent validity was high for the LCQ and QoL-B, moderate for the CAT, and very low for the SGRQ demonstrating all PROMs sufficient convergent validity (Table 4).

**Table 4:** Results on convergent and discriminative validity of patient-reported outcome measures for Spanish-speaking people with bronchiectasis

PROMs	Hypothesis testing											
	Convergent validity						Discriminative validity					
	n	PROM / Clinical outcome	Synthesised results	RoB	Rating	QoE	n	Severity score / Clinical outcome	Synthesised results^£^	RoB	Rating	QoE
LCQ	259	Total LCQ—total SGRQ Total LCQ—domains of SGRQ	r = -0.66 ^✓^ r = from -0.52 to -0.68 ^✓^	V	(+)	High	255	Total LCQ—FACED	Mild vs moderate = ES = 0.4^✓^ Mild vs Severe = ES = 0.74 and > MID (1.3 points) ^✓^	V	(+)	High
		Physical domain of LCQ—domains of SGRQ Psychological domain of LCQ—domains of SGRQ Social domain of LCQ—domains of SGRQ	r = from -0.54 to -0.65^✓^ r = from -0.45 to -0.54^✓^ r = from -0.52 to -0.66^✓^					Total LCQ—BSI	Mild vs moderate ES = 0.54^✓^ Mild vs severe ES = 0.77 and > MID (1.3 points)^✓^			
QoL-B	207	Physical functioning—Total SGRQ Role functioning– Total SGRQ Vitality– Total SGRQ Emotional functioning—Total SGRQ Social functioning—Total SGRQ Treatment burden—Total SGRQ Health perceptions—Total SGRQ Respiratory symptoms—Total SGRQ	r = -0.81^✓^ r = -0.77^✓^ r = -0.67^✓^ r = -0.64^✓^ r = -0.53^✓^ r = -0.34 r = -0.68^✓^ r = -0.69^✓^	V	(+)	High	207	Domains of QoL-B—Age	r = ranges from -0.08 to -0.45^✓^	D	(+)	Low^&^
								Domains of QoL-B—Lung function (FEV_1_)	r = ranges from 0.11 to 0.41^✓^			
								Domains of QoL-B—Exacerbations	r = ranges from -0.07 to -0.25			
								Domains of QoL-B—Disease extent (Bhalla score)	r = ranges from 0.16 to 0.45^✓^			
		Physical functioning—domains of SGRQ Role functioning– domains of SGRQ Vitality—domains of SGRQ Emotional functioning—domains of SGRQ Social functioning—domains of SGRQ Treatment burden—domains of SGRQ Health perceptions—domains of SGRQ Respiratory symptoms—domains of SGRQ	r = from -0.56 to -0.78^✓^ r = from -0.48 to -0.77^✓^ r = from -0.50 to -0.63^✓^ r = from -0.40 to -0.64^✓^ r = from -0.30 to -0.58^✓^ r = from -0.23 to -0.37 r = from -0.43 to -0.70^✓^ r = from -0.54 to -0.65^✓^					Domains of QoL-B—Respiratory symptoms (sputum production)	r = ranges from -0.18 to -0.48^✓^			
								Domains of QoL-B—Respiratory symptoms (dyspnoea)	r = ranges from -0.19 to -0.60^✓^			
								Domains of QoL-B—Comorbidity (Charlson comorbidity index)	r = ranges from -0.03 to -0.19			
CAT	96	CAT—BHQ	r = -0.70 ^✓^	V	(+)	Moderate^*^	96	FACED	r = 0.25	V	(+)	Moderate^*^
		CAT—Total SGRQ	r = 0.75^✓^					E-FACED	r = 0.26			
		CAT -domains of SGRQ	r = ranges from 0.59 to 0.67^✓^					BSI	r = 0.22			
		CAT—domains of QoL-B	r = ranges from -0.68 to 0.52^✓^									
SGRQ	102	Total SGRQ—Disease extent	r = 0.33^✓^	I	(+)	Very low^@^	102	Total SGRQ -Disease extent	ES ~ 0.62^✓^	D	(+)	Low^&^
		Total SGRQ -Airway infection (*P. aeruginosa*)	r = 0.35^✓^					Total SGRQ—Airway infection (*P. aeruginosa*)	ES ~ 0.87^✓^			
		Total SGRQ -Exacerbations	r = 0.20					Total SGRQ- Exacerbations	ES ~ 0.77^✓^			
		Total SGRQ -Lung function (FEV_1_)	r = 0.59^✓^					Total SGRQ—Lung function (FEV_1_)	ES ~ 1.12^✓^			
		Total SGRQ -Respiratory symptoms (sputum production)	r = 0.47^✓^					Total SGRQ—Respiratory symptoms (sputum production)	ES ~ 0.73^✓^			
		Total SGRQ—Respiratory symptoms (dyspnoea)	r = 0.47^✓^					–	–			
		Respiratory symptoms (cough)	r = 0.32^✓^					–	–			

PROMs, patient-reported outcomes measures; LCQ: Leicester Cough Questionnaire; QoL-B: Quality of Life Questionnaire for Bronchiectasis; CAT: COPD Assessment Test; SGRQ: Saint George’s Respiratory Questionnaire; RoB: Risk of Bias; V: very good; A: Adequate; D: Doubtful; I: Inadequate; FEV_1_: Forced expiratory volume in one second; HRCT: High resolution computed tomography; BMI: body mass index; BSI: Bronchiectasis Severity Index; QoE, quality of evidence; £ Effect sizes were estimated from study data using pooled SD (weighted when sample sizes were available, unweighted when not) when not reported directly in the original articles; ^✓^ pre-specified hypothesis was confirmed as anticipated; (+) sufficient. *: -1 imprecision (sample size 50–100); @:-3 Risk of Bias extremely serious; &: -2 Risk of Bias serious

Respiratory symptoms (cough) r = 0.32*

The CAT and LCQ tools [23, 24] were developed after the introduction of validated disease severity scores in bronchiectasis. These studies utilised the BSI, FACED, and e-FACED scales to assess the discriminative validity. Conversely, earlier studies (QoL-B and SGRQ tools [22, 25]) investigated discriminative validity through clinical parameters associated with disease severity, including respiratory symptoms, *Pseudomonas aeruginosa* infection, lung function, exacerbations, radiological disease extent, and airway infection. Among these PROMs examined, all were rated as sufficient discriminative validity. However, the LCQ demonstrating high-quality evidence of discriminative validity (RoB: very good). The CAT tool showed moderate quality of evidence (RoB: very good), while the QoL-B and SGRQ instruments exhibited low quality of evidence of discriminative validity (RoB: doubtful) (Table 4).

#### Reliability

Internal consistency was evaluated in all included studies [22–25], with Cronbach's alpha values ranging from 0.70 to 0.91. Although all studies demonstrated a low risk of bias, according to COSMIN guidelines, structural validity must be assessed as a prerequisite to justify the interpretation of internal consistency. Structural validity, which was evaluated only for the SGRQ and rated as very low quality, is essential to confirm unidimensionality. Due to this, the internal consistency for all instruments was rated as indeterminate, and the quality of evidence for internal consistency could not be determined [18, 20] (Table 5).

**Table 5:** Results on reliability of patient-reported outcome measures for Spanish-speaking people with bronchiectasis

PROM	Reliability														
	Internal consistency					Test–retest reliability					Measurement error				
	n	Result	RoB	Rating^**£**^	QoE^¥^	n	Result	RoB	Rating	QoE	n	Result	RoB	Rating	QoE
LCQ	259	α (total) = 0.91 α (physical) = 0.87 α (phycological) = 0.87 α (social) = 0.86	V	(?)		199	ICC (total) = 0.84 (0.79–0.87) ICC (physical) = 0.87 (0.84–0.90) ICC (phycological) = 0.82 (0.77–0.86) ICC (social) = 0.79 (0.73–0.84)	V	(+)	High	199	Mean difference = -0.10 Limits of agreement = -4.8 to 4.6	V	(−)	High
QoL-B	207	α (physical functioning) = 0.91 α (rol functioning) = 0.84 α (vitality) = 0.82 α (emotional functioning) = 0.84 α (social functioning) = 0.70 α (burden treatment) = 0.72 α (health perceptions) = 0.71 α (respiratory symptoms) = 0.87	V	(?)		161	ICC (physical functioning) = 0.88 ICC (rol functioning) = 0.86 ICC (vitality) = 0.78 ICC (emotional functioning) = 0.86 ICC (social functioning) = 0.78 ICC (burden treatment) = 0.68 ICC (health perceptions) = 0.83 ICC (respiratory symptoms) = 0.83	A	(+)	Moderate^#^		NR			
CAT	96	α (total) = 0.86	V	(?)		96	ICC Total = 0.95 (0.92–0.97)	A	(+)	Low^*#^	96	- No numerical values	V	(−)	Moderate^*^
SGRQ	102	α (total) = 0.90 α (symptoms) = 0.81 α (activity) = 0.87 α (impact) = 0.8	V	(?)			NR					NR			

LCQ, Leicester Cough Questionnaire; QoL-B, Quality of Life Questionnaire for Bronchiectasis; CAT, COPD Assessment Test; SGRQ, Saint George's Respiratory Questionnaire; RoB, Risk of Bias; V, very good; A, Adequate; ICC, Intraclass correlation coefficient; NR, not reported; £ Internal consistency was rated as indeterminate because evidence for structural validity was either of very low quality or not reported for the remaining PROMs, in accordance with COSMIN standards. ¥ No GRADE evaluation should be applied when the rating is indeterminate.; (+) sufficient; (−) insufficient; (?) indeterminate *: -1 imprecision (sample size 50–100); #: -1 Risk of Bias serious

Test–retest reliability was evaluated in all instruments except the SGRQ study. The methodological RoB varied across studies, classified from adequate for the QoL-B and CAT to very good for the LCQ. Intraclass correlation coefficients ranged from 0.68 to 0.95. The quality of evidence for this measurement property was high for the LCQ, moderate for the QoL-B and low for the CAT instrument (Table 5). The measurement error property was evaluated only for the LCQ and CAT instruments. The quality of evidence was high for the LCQ and moderate for the CAT. Both instruments were classified as having very good methodology according to the RoB assessment. However, they were rated as insufficient because the limits of agreement exceeded the minimal important change values (Table 5).

#### Responsiveness

Responsiveness was assessed for the LCQ, QoL-B and CAT instruments by comparing scores obtained in stable condition with those at the onset of an exacerbation [22–24]. Minimum important differences were reported for the CAT (3 points) and the respiratory domain of the QoL-B (8.2 points). All instruments were rated as having sufficient responsiveness, with the QoL-B and CAT classified as very good and the LCQ as doubtful according to the RoB assessment. The quality of evidence for responsiveness was moderate for the QoL-B and CAT and very low for the LCQ (Table 6).

**Table 6:** Results on responsiveness of patient-reported outcome measures for Spanish-speaking people with bronchiectasis

PROM	Responsiveness											
	Comparison with other outcome measurement instruments/clinical outcomes						Comparison before and after intervention / exacerbation					
	n	PROM/clinical outcome	Synthesised results	RoB	Rating	Quality of evidence	n	PROM/clinical outcome/domain	Synthesised results^£^	RoB	Rating	Quality of evidence
LCQ		NR					95	Total score	15.13 (4.06) *vs.* 12.24 (4.64); ES = 0.66^✓^	D	(+)	Very low^*&^
								Physical score	4.82 (1.44) *vs.* 3.89 (1.49); ES = 0.64^✓^			
								Psychological score	4.97 (1.45) *vs.* 4.09 (1.64); ES = 0.57^✓^			
								Social score	5.32 (1.47) *vs.* 4.24 (1.71); ES = 0.68^✓^			
QoL-B	80	Respiratory symptoms—sputum volume (mL)	r = 0.34^✓^	I	(?)	Very low^*@^	80	Physical functioning	51.6 (29.1) *vs.* (38.4 ± 29.3); ES = 0.45^✓^	V	(+)	High
								Role functioning	68.0 (25.8) *vs.* 51.1 (28.4); ES = 0.62^✓^			
								Vitality	54.4 (24.7) *vs.* 39.4 (29.6); ES = 0.55^✓^			
								Emotional functioning	69.3 (27.4) *vs.* 65.9 (26.0); ES = 0.13			
								Social functioning	68.9 (28.2) *vs.* 60.4 (29.0): ES = 0.32^✓^			
								Treatment burden	67.6 (24.8) *vs.* 61.8 (24.0); ES = 0.24			
								Health perception	43.0 ± 19.5 *vs.* 32.2 ± 22.4; ES = 0.52^✓^			
								Respiratory symptoms	69.9 (18.9) *vs.* 52.2 (21.3); ES = 0.88^✓^ MID (distribution-based methods) = 8.2			
CAT		NR					57	MID (distribution-based methods) = 3		V	(+)	Moderate^*^
SGRQ		NR						NR				

LCQ, Leicester Cough Questionnaire; QoL-B, Quality of Life Questionnaire for Bronchiectasis; CAT, COPD Assessment Test; SGRQ, Saint George's Respiratory Questionnaire; RoB, Risk of Bias; D, Doubtful; I, Inadequate; NR, no reported; MCID, Minimum clinical important difference; NR, not reported; £ Effect sizes were estimated from study data using pooled SD (weighted when sample sizes were available, unweighted when not) when not reported directly in the original articles;^✓^ pre-specified hypothesis was confirmed as anticipated; (?) indeterminate; (+) sufficient; *: -1 imprecision (sample size 50–100); @: -3 Risk of Bias extremely serious; &: -2 Risk of Bias serious

Additionally, responsiveness was also evaluated for the respiratory symptoms domain of the QoL-B by comparing changes between stable and exacerbation clinical conditions with another outcome measure, specifically change in sputum volume [22]. Its classification according to the RoB assessment was inadequate, resulting in an indeterminate rating and demonstrating very low quality of evidence (Table 6).

## Discussion

The aim of this review was to synthesise the measurement properties of the PROMs available for Spanish-speaking people with bronchiectasis. Four different PROMs [22–25] were identified and described: two questionnaires evaluate quality of life with different subdomains, while the other questionnaires explore symptoms that impact daily life. Convergent and discriminant validity, along with reliability, were the most extensively explored measurement properties in the validation process of these PROMs for Spanish-speaking people with bronchiectasis, with the quality of evidence ranging from very low to high quality. In contrast, structural validity, cross-sectional validity and responsiveness were the least studied measurement properties and showed the poorest quality of evidence, limiting the comparability of their scores across different cultural contexts and their applicability for evaluating treatment response. These findings underscore the need for further research in this area.

To reduce disparities and ensure uniform accessibility to PROMs regardless of native language, validated translation processes, cultural adaptation, and subsequent measurement property analyses are strongly recommended to enhance usability for non-English-speaking populations, particularly in clinical practice [29]. This review demonstrates that although the number of PROMs developed for bronchiectasis has increased [13], their validation in other languages remains scarce, with only four PROMs available for Spanish-speaking people. Some PROMs, like the Bronchiectasis Health Questionnaire [28], have been translated into Spanish, but these translations often relied solely on linguistic experts without patient involvement at any stage of the adaptation process. This raises concerns regarding the cultural relevance and linguistic accuracy of the translated instruments and indicates that best practices for instrument for adaptation were not fully met [18, 20].

Moreover, the PROMs included in this study were translated using exclusively populations from Spain; thus, for Spanish-speakers from other regions, a cultural adaptation may be required. Content validity, structural validity and cross-cultural validation of PROMs presents a significant challenge due to the time-consuming process of recruiting participants and the complex statistical analyses required, such as confirmatory factor analysis [21]. This may explain why only a limited number of accessible PROMs for Spanish-speaking people were identified. Content validity based on the authors' opinions appears to demonstrate sufficient relevance, comprehensiveness, and comprehensibility, albeit with low or very low quality of evidence. Finally, only one PROM was initially designed for patients with bronchiectasis [22], indicating that the remaining instruments have required prior evaluation of their measurement properties in this specific population before use.

Hypothesis testing was assessed by the convergent and discriminative validity. Most of the studies [22–24] provided evidence of sufficient convergent validity with a quality of evidence of high or moderate. Surprisingly, the SGRQ [25], traditionally regarded as the gold-standard comparator for convergent validity in bronchiectasis, was the PROM that received the lowest quality of evidence rating in this section. Since the SGRQ predates the other PROMs, authors selected clinical outcome measures as reference standards rather than other PROMs. This selection may have negatively impacted the quality of evidence.

A similar situation has been observed with respect to discriminative validity. Evidence indicates that the CAT and LCQ demonstrated high-quality with a low RoB for discriminant validity, primarily because these instruments used validated severity score (BSI, FACED, and E-FACED) in their assessments. In contrast, the evaluations of the SGRQ and QoL-B were conducted before the availability of these validated severity scores for bronchiectasis. Consequently, the findings related to the SGRQ and QoL-B should be interpreted with caution. Given these chronological discrepancies, future studies are encouraged to re-evaluate the validity of these instruments, considering the COSMIN guidelines and the currently available disease severity scores for bronchiectasis.

Overall, internal consistency must be rated as indeterminate despite the low risk of bias observed across all PROMs due to either insufficient structural validity evidence or only very low-quality structural validity data [18]. For test–retest reliability, the evidence from the included studies indicates moderate to high quality for the LCQ and QoL-B instruments. In contrast, the low quality of evidence for the CAT test–retest reliability is primarily due to a limited sample size. These findings should be interpreted with caution, as COSMIN recommends that internal consistency be supported by adequate structural validity, which was not evaluated in these PROMs. Additionally, test–retest reliability was not assessed in the SGRQ study, and measurement error was not evaluated for either the QoL-B or SGRQ. Test–retest reliability and measurement error evaluation are essential parameters for determining the suitability of PROMs for longitudinal monitoring [30]. They also facilitate the interpretation of whether changes in PROM scores reflect true changes in patient clinical status, rather than measurement imprecision [30]. Therefore, it is encouraged that future studies evaluate all reliability parameters to support the selection of the most appropriate PROMs in bronchiectasis.

Responsiveness was one of the least studied measurement properties, with evidence indicating poor quality of evidence. The LCQ, QoL-B and CAT instruments [22–24] assessed responsiveness by comparing results from the same sample at two different time points: during clinical stability and during an acute exacerbation. However, only the QoL-B instrument compared the changes observed between these two time points with changes in other clinical outcomes. Therefore, none of these instruments evaluated responsiveness following a therapeutic intervention, which limits their utility in assessing the effectiveness of interventions. Additionally, the MID was reported for both the respiratory symptoms domain of QoL-B and CAT; however, in both cases, the MID was calculated using a distribution-based approach. This limits the interpretation of the MID as a clinically meaningful magnitude for patients. Addressing both of these limitations should be a priority for future research. Doing so will provide valuable insights for selecting the most suitable PROMs for use in clinical practice and clinical trials, while ensuring that patients’ perspectives are appropriately considered.

Among the PROMs included in this review, the QoL‑B (especially the respiratory symptoms domain) and the SGRQ are the most commonly used to monitor and evaluate treatment response in bronchiectasis [16, 31]. For Spanish-speaking populations, our findings indicate that interpretation of change appears more straightforward with the QoL‑B respiratory domain, which has an established MID and stronger evidence for reliability and convergent validity than the SGRQ. The QoL‑B is also quicker to administer and specifically developed for bronchiectasis. The LCQ effectively assesses cough with high-quality evidence for reliability and hypothesis testing, though its Spanish version lacks an established MID. The CAT, commonly used in COPD, was recently evaluated in bronchiectasis and, due to its brevity and ease of use, may become a practical tool for Spanish-speaking patients despite evidence suggest having moderate-quality evidence globally.

### Strengths and limitations

A major strength of this systematic review lies in its rigorous application of the COSMIN taxonomy and guidelines to assess both the methodological quality of the included studies and the quality of the measurement properties of the various PROMs. This approach supports the synthesis and comparison of findings across different PROMs and helps identify measurement properties that are either under-examined or assessed using suboptimal methods. As a result, it highlights areas for improvement and offers direction for future research.

In contrast, a key limitation of this systematic review was the limited availability of information needed to evaluate the cross-cultural validity of the PROMs. In particular, the translated versions used for Spanish-speaking populations were often validated in the context of other respiratory diseases, such as COPD [26, 27]. Consequently, information regarding cross-cultural validity had to be obtained from the original validation studies of these PROMs for Spanish-speaking populations [26, 27].

Another limitation is the small number of studies identified, highlighting the lack of accessible monitoring tools for non-English-speaking individuals with bronchiectasis. Since our findings focus on Spanish-speaking populations, they should not be generalized to other languages due to linguistic, cultural, and healthcare differences affecting PROM performance. Addressing this gap will require future systematic reviews in other languages through coordinated international efforts to build a global evidence base and ensure rigorous language-specific psychometric validation. This approach would enhance comparability across settings and reduce inequities in patient care. Finally, despite COSMIN guidance, some PROMs are still used without validated translations and cultural adaptations, potentially compromising result interpretation.

## Conclusion

Few PROMs are currently available for Spanish-speaking adults with bronchiectasis. Evidence from the included studies suggests sufficient convergent and discriminative validity and test–retest reliability for these PROMs in the populations studied. However, content validity, structural validity, cross-cultural validity and responsiveness were rarely explored, limiting confidence that these instruments fully capture the intended constructs, accurately reflect changes across diverse populations, and support interpretation of treatment responses or clinical deterioration. Notably, the lack or very low-quality evidence for structural validity also impacts the interpretation of internal consistency, which must be rated as indeterminate. Further research in this area is essential to enhance the accessibility of PROMs for non-English speakers, improve usability in clinical practice, and reduce disparities.

## Data Availability

The search strategies and PRISMA flow diagram supporting this review are available in the manuscript/ supplementary materials. Extracted data are available from the corresponding author [BH-C] upon reasonable request.

## References

[1] Long, M. B., Chotirmall, S. H., Shteinberg, M., & Chalmers, J. D. (2024). Rethinking bronchiectasis as an inflammatory disease. *The Lancet Respiratory Medicine,**12*(11), 901–914. 38971168 10.1016/S2213-2600(24)00176-0

[2] Im, Y., Chalmers, J. D., & Choi, H. (2025). Disease severity and activity in bronchiectasis: a paradigm shift in bronchiectasis management. *Tuberculosis Respir Dis,**88*(1), 109–119. 10.4046/trd.2024.0120 PMC11704736 39218441

[3] Nigro, M., Laska, I. F., Traversi, L., Simonetta, E., & Polverino, E. (2024). Epidemiology of bronchiectasis. *European Respiratory Reviews*. 10.1183/16000617.0091-2024 10.1183/16000617.0091-2024 PMC11462313 39384303

[4] Chalmers, J. D., Mall, M. A., McShane, P. J., Nielsen, K. G., Shteinberg, M., Sullivan, S. D., et al. (2024). A systematic literature review of the clinical and socioeconomic burden of bronchiectasis. *European Respiratory Reviews*. 10.1183/16000617.0049-2024 10.1183/16000617.0049-2024 PMC11372470 39231597

[5] Roberts, J. M., Goyal, V., Kularatna, S., Chang, A. B., Kapur, N., Chalmers, J. D., et al. (2023). The economic burden of bronchiectasis: a systematic review. *Chest,**164*(6), 1396–1421. 37423293 10.1016/j.chest.2023.06.040

[6] de la Rosa Carrillo, D., Navarro Rolon, A., Giron Moreno, R. M., Montull Veiga, B., Olveira Fuster, C., Padilla Galo, A., et al. (2018). Cost of hospitalizations due to exacerbation in patients with non-cystic fibrosis bronchiectasis. *Respiration,**96*(5), 406–416. 29996130 10.1159/000489935

[7] Aliberti, S., Lonni, S., Dore, S., McDonnell, M. J., Goeminne, P. C., Dimakou, K., et al. (2016). Clinical phenotypes in adult patients with bronchiectasis. *European Respiratory Journal,**47*(4), 1113–1122. 26846833 10.1183/13993003.01899-2015

[8] Watson, K. E., Lee, A. L., Dwyer, T. J., & McKeough, Z. J. (2024). Applying the treatable traits approach in bronchiectasis-A scoping review of traits, measurements and treatments implemented by allied health professionals and nurses. *Respiratory Medicine,**222*, Article 107503. 38141863 10.1016/j.rmed.2023.107503

[9] Alcaraz-Serrano, V., Gimeno-Santos, E., Scioscia, G., Gabarrus, A., Navarro, A., Herrero-Cortina, B., et al. (2020). Association between physical activity and risk of hospitalisation in bronchiectasis. *European Respiratory Journal*. 10.1183/13993003.02138-2019 32184321 10.1183/13993003.02138-2019

[10] Boaventura, R., Sibila, O., Agusti, A., & Chalmers, J. D. (2018). Treatable traits in bronchiectasis. *European Respiratory Journal*. 10.1183/13993003.01269-2018 30190263 10.1183/13993003.01269-2018

[11] Carrozzino, D., Patierno, C., Guidi, J., Berrocal Montiel, C., Cao, J., Charlson, M. E., et al. (2021). Clinimetric criteria for patient-reported outcome measures. *Psychotherapy and Psychosomatics,**90*(4), 222–232. 34038901 10.1159/000516599

[12] Spinou, A., Fragkos, K. C., Lee, K. K., Elston, C., Siegert, R. J., Loebinger, M. R., et al. (2016). The validity of health-related quality of life questionnaires in bronchiectasis: a systematic review and meta-analysis. *Thorax,**71*(8), 683–694. 26869589 10.1136/thoraxjnl-2015-207315

[13] McLeese, R. H., Spinou, A., Alfahl, Z., Tsagris, M., Elborn, J. S., Chalmers, J. D., et al. (2021). Psychometrics of health-related quality of life questionnaires in bronchiectasis: a systematic review and meta-analysis. *European Respiratory Journal*. 10.1183/13993003.00025-2021 33888521 10.1183/13993003.00025-2021 PMC8581652

[14] Castellvi P, Ferrer M, Alonso J, en nombre del Comite Cientifico de Biblio PRO (2013) The patient-reported outcomes in research: definition, impact, classification, measurement and assessment. Med Clin (Barc) 141(8):358–65 10.1016/j.medcli.2013.07.013 24060501

[15] Sibila, O., Laserna, E., Shoemark, A., Perea, L., Bilton, D., Crichton, M. L., et al. (2022). Heterogeneity of treatment response in bronchiectasis clinical trials. *European Respiratory Journal*. 10.1183/13993003.00777-2021 34675045 10.1183/13993003.00777-2021

[16] Gao, Y. H., Abo Leyah, H., Finch, S., Lonergan, M., Aliberti, S., De Soyza, A., et al. (2020). Relationship between symptoms, exacerbations, and treatment response in bronchiectasis. *American Journal of Respiratory and Critical Care Medicine,**201*(12), 1499–1507. 32097051 10.1164/rccm.201910-1972OC

[17] Crichton, M. L., Dudgeon, E. K., Shoemark, A., & Chalmers, J. D. (2021). Validation of the bronchiectasis impact measure (BIM): a novel patient-reported outcome measure. *European Respiratory Journal*. 10.1183/13993003.03156-2020 33214211 10.1183/13993003.03156-2020

[18] Mokkink, L. B., Elsman, E. B. M., & Terwee, C. B. (2024). COSMIN guideline for systematic reviews of patient-reported outcome measures version 2.0. *Quality of Life Research,**33*(11), 2929–2939. 39198348 10.1007/s11136-024-03761-6 PMC11541334

[19] Elsman, E. B. M., Mokkink, L. B., Terwee, C. B., Beaton, D., Gagnier, J. J., Tricco, A. C., et al. (2024). Guideline for reporting systematic reviews of outcome measurement instruments (OMIs): PRISMA-COSMIN for OMIs 2024. *Quality of Life Research,**33*(8), 2029–2046. 38980635 10.1007/s11136-024-03634-y PMC11286641

[20] Mokkink LB, Elsman E, Terwee CB (2024) COSMIN manual for systematic reviews of Patient-Reported Outcome Measures (PROMs): version 2.0. Amsterdam: Amsterdam UMC 10.1007/s11136-024-03761-6 PMC11541334 39198348

[21] Mokkink, L. B., de Vet, H. C. W., Prinsen, C. A. C., Patrick, D. L., Alonso, J., Bouter, L. M., et al. (2018). COSMIN risk of bias checklist for systematic reviews of patient-reported outcome measures. *Quality of Life Research,**27*(5), 1171–1179. 29260445 10.1007/s11136-017-1765-4 PMC5891552

[22] Olveira, C., Olveira, G., Espildora, F., Giron, R. M., Munoz, G., Quittner, A. L., et al. (2014). Validation of a quality of life questionnaire for bronchiectasis: psychometric analyses of the Spanish QOL-B-V3.0. *Quality of Life Research,**23*(4), 1279–1292. 24142190 10.1007/s11136-013-0560-0

[23] Munoz, G., Buxo, M., de Gracia, J., Olveira, C., Martinez-Garcia, M. A., Giron, R., et al. (2016). Validation of a Spanish version of the Leicester Cough Questionnaire in non-cystic fibrosis bronchiectasis. *Chronic Respir Dis,**13*(2), 128–136. 10.1177/1479972316632005 PMC5734594 26902541

[24] De la Rosa, C. D., Olveira, C., Garcia-Clemente, M., Giron-Moreno, R. M., Nieto-Royo, R., Navarro-Rolon, A., et al. (2020). COPD assessment test in bronchiectasis: minimum clinically important difference and psychometric validation: a prospective study. *Chest,**157*(4), 824–833. 10.1016/j.chest.2019.08.1916 31446064

[25] Martinez Garcia, M. A., Perpina Tordera, M., Roman Sanchez, P., & Soler Cataluna, J. J. (2005). [Internal consistency and validity of the Spanish version of the St. George’s respiratory questionnaire for use in patients with clinically stable bronchiectasis]. *Archivos de Bronconeumología,**41*(3), 110–117. 15766462 10.1016/s1579-2129(06)60410-2

[26] Agusti, A., Soler, J. J., Molina, J., Munoz, M. J., Garcia-Losa, M., Roset, M., et al. (2012). Is the CAT questionnaire sensitive to changes in health status in patients with severe COPD exacerbations? *COPD,**9*(5), 492–498. 22958111 10.3109/15412555.2012.692409

[27] Ferrer, M., Alonso, J., Prieto, L., Plaza, V., Monso, E., Marrades, R., et al. (1996). Validity and reliability of the St George’s Respiratory Questionnaire after adaptation to a different language and culture: the Spanish example. *European Respiratory Journal,**9*(6), 1160–1166. 8804932 10.1183/09031936.96.09061160

[28] Spinou, A., Siegert, R. J., Guan, W. J., Patel, A. S., Gosker, H. R., Lee, K. K., et al. (2017). The development and validation of the Bronchiectasis Health Questionnaire. *European Respiratory Journal*. 10.1183/13993003.01532-2016 28495688 10.1183/13993003.01532-2016

[29] Sousa, V. D., & Rojjanasrirat, W. (2011). Translation, adaptation and validation of instruments or scales for use in cross-cultural health care research: a clear and user-friendly guideline. *J Evaluat Clin Pract,**17*(2), 268–274. 10.1111/j.1365-2753.2010.01434.x 20874835

[30] Mokkink, L. B., Boers, M., van der Vleuten, C. P. M., Bouter, L. M., Alonso, J., Patrick, D. L., et al. (2020). COSMIN risk of bias tool to assess the quality of studies on reliability or measurement error of outcome measurement instruments: a Delphi study. *BMC Medical Research Methodology,**20*(1), 293. 33267819 10.1186/s12874-020-01179-5 PMC7712525

[31] Gao, Y. H., Zheng, H. Z., Lu, H. W., Li, Y. Y., Feng, Y., Gu, S. Y., et al. (2024). Quality-of-life bronchiectasis respiratory symptom scale predicts the risk of exacerbations in adults with bronchiectasis: a prospective observational study. *Ann Am Thoracic Soc,**21*(3), 393–401. 10.1513/AnnalsATS.202302-133OC 37962906

